# Efficacy of Combination Therapies for Autoimmune Hepatitis: A Systematic Review and Meta-Analysis

**DOI:** 10.7759/cureus.60049

**Published:** 2024-05-10

**Authors:** Essam Rashad, Mustafa M Moazam, Raheel Chaudhry, Noha El Eraky, Muhammad Sohail S Mirza, Farhana Nazmin

**Affiliations:** 1 Hospital Medicine, Parkview Regional Medical Center, Fort Wayne, USA; 2 Psychiatry, Texas Tech University Health Sciences Center El Paso, El Paso, USA; 3 Medicine, Baylor College of Medicine, Houston, USA; 4 Radiology, St Vincent's University Hospital, Dublin, IRL; 5 Internal Medicine, Shandong University School of Medicine, Jinan, CHN; 6 Psychiatry, BronxCare Health System (BCHS), Bronx, USA

**Keywords:** azathioprine, prednisolone, efficacy, combination therapy, autoimmune hepatitis

## Abstract

Autoimmune hepatitis (AIH) is a hepatocellular disorder thought to be caused by an immune system that cannot tolerate autoantigens specific to hepatocytes. This study aims to evaluate the efficacy of using corticosteroids (prednisolone and azathioprine) as a combination therapy in treating AIH.

This study aims to synthesize and analyze existing evidence to inform clinical practices concerning the overall clinical efficacy of this treatment approach in managing AIH.

A comprehensive search was conducted across multiple online databases and search engines, including PubMed, Google Scholar, ScienceDirect, Medline, and Embase. RevMan 5.4 software was used for meta-analysis, with forest plots created for each outcome.

Thirteen studies were included in this systematic review and meta-analysis. The results indicate that the combination of prednisolone and azathioprine for treating AIH leads to less recurrence and better disease control.

## Introduction and background

The hepatocellular disorder known as autoimmune hepatitis (AIH) is thought to be caused by an immune system that is unable to tolerate autoantigens that are unique to hepatocytes [1]. Like other autoimmune diseases, AIH is a common illness that affects people of many racial and ethnic backgrounds, both in children and adults [2,3]. AIH progresses at different rates in untreated individuals. Individuals who use immunosuppressive medications to induce remission have an extremely good prognosis. On the other hand, lifesaving orthotopic liver transplantation (OLT) is required for patients who present with acute liver failure (ALF) and those who exhibit or experience severe complications related to cirrhosis or hepatocellular carcinoma [4]. Jan Waldenström described the typical clinical features of patients with AIH in September 1950 [5]. The patient was a young woman who had elevated gamma globulin levels and hepatic dysfunction. Since then, AIH diagnostic standards have been developed and improved [6,7]. AIH can be diagnosed in people of all ages and genders, and the condition is increasingly recognized as a global health issue [8,9]. In Alaska Natives, the prevalence is very high at 42.9 cases per 100,000 people [10]. According to early reports, the age distribution of AIH was bimodal, with two peaks: one between the ages of 40 and 50, and the other between the ages of 10 and 30 [11]. However, biases in reporting from tertiary referral centers might have affected this finding. Furthermore, the disorder is now more widely recognized, with cases found in people of all ages, from newborns to those in their eighties [12-14]. Regional and ethnic differences significantly impact the clinical presentation, course, and prognosis of AIH. Northern European descent shows a prevalence of 38%, while the African-American population shows a 56% to 85% prevalence [15,16]. In the United States, people have early onset disease and respond to powerful immunosuppressive agents, while it is quite the opposite for the Japanese population [17,18].

The clinical side of AIH is challenging. There is a need to evaluate the efficacy of different treatment options. This study aims to study the effect of corticosteroids (prednisolone and azathioprine) in the treatment of AIH.

## Review

Methodology

Search Strategy

Throughout the study, ethical considerations such as user privacy and confidentiality were carefully addressed (Table 1).

**Table 1:** PICO table of the systematic review.

Population (P)	Patients with autoimmune hepatitis
Intervention (I)	Combination therapies for autoimmune hepatitis
Comparison (C)	Standard or individualized therapies (comparator may vary based on the studies included in the review)
Outcome (O)	Efficacy in managing autoimmune hepatitis, considering factors such as disease remission, improvement in liver function, and reduction in adverse effects.

Inclusion Criteria

Inclusion criteria for the study encompassed patients diagnosed with AIH, consideration of combination therapies targeting AIH, publications in peer-reviewed journals, and research studies with full-text articles.

Exclusion Criteria

Exclusion criteria consisted of studies that did not primarily focus on AIH, non-peer-reviewed publications, including conference abstracts and posters, research with insufficient or incomplete data, and studies focusing exclusively on pediatric populations or data derived solely from pediatric sources (Table 2).

**Table 2:** Eligibility criteria.

Inclusion criteria	Exclusion criteria
Patients with autoimmune hepatitis	Studies not focusing on autoimmune hepatitis
Combination therapies targeting autoimmune hepatitis	Non-peer-reviewed publications, including conference abstracts and posters
Publications in peer-reviewed journals	Research with insufficient or incomplete data
Research studies with full-text articles	Research on pediatric populations or data derived solely from pediatric sources

Information Sources

Several digital databases containing relevant literature such as PubMed, Google Scholar, ScienceDirect, and others were utilized. In addition, independent journals and other independent sources were included.

Search Strategy

The strategy is refined by including synonyms and variations of these terms in the search using Boolean operators (AND, OR). Search filters are used to limit the results to human-subject studies published in peer-reviewed journals. Furthermore, reference lists of relevant articles and grey literature are manually searched to identify potential studies that would have been missed by electronic searches. The search strategy followed was ("autoimmune hepatitis" OR "AIH" OR "autoimmune liver disease") AND ("combination therapy" OR "combined treatment" OR "multimodal therapy") AND ("efficacy" OR "effectiveness" OR "outcome"). The search strategy consists of a systematic and comprehensive search strategy that aims to be inclusive and comprehensive, capturing a diverse range of studies while remaining relevant to the research question.

Selection Process

The articles were double-screened. This review yielded a list of possible papers. Any disagreements were settled through discussion and the senior author's decision. Because the outcome measures in the meta-analysis studies were similar, they were chosen. The meta-analysis excluded studies that lacked sufficient comparable data or had poor methodological quality.

Data Collection

We searched peer-reviewed journals and publications for literature that met the inclusion criteria. To reduce the risk of publication bias, peer-reviewed journals were investigated after a thorough review of the literature. We worked to *include* or *exclude* eligible studies. Studies that failed to meet the screening criteria were labeled *exclusion* or *dispute*. Before eliminating a study from the literature, exclusion reasons were presented. It was sometimes caused by an interaction of exclusionary factors.

Quality Assessment

The quality assessment included three broad categories of questions: (1) Were the findings of the study validated? (2) What were the results? (3) Are the findings of the study applicable locally? Eleven questions for quality assessment were answered after careful consideration of study designs and findings. The questions were answered with *Yes*, *No*, and *Can't tell*. If you answered *Yes* to the first question, you should answer the remaining questions. There is some overlap in the questions. The explanations for the answers, as well as comments from researchers, have been included in the Results section.

Results

Data Items

**Figure 1:**
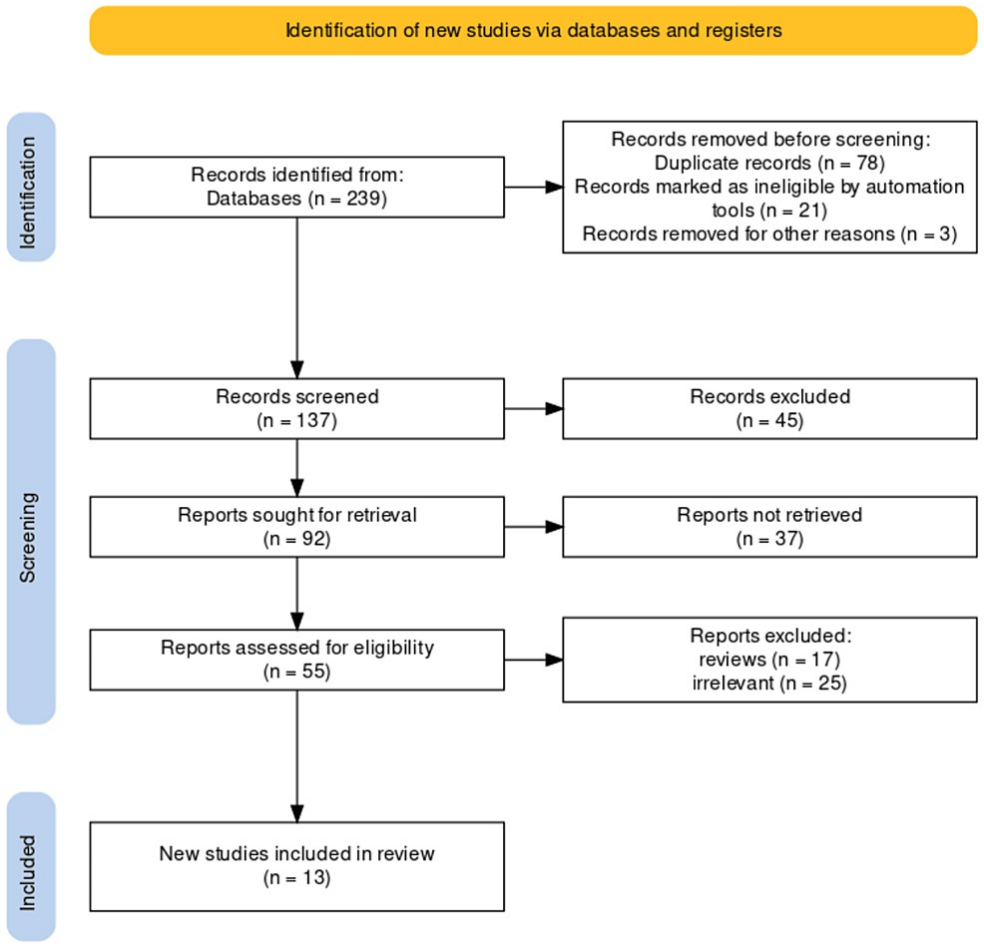
PRISMA flowchart of included studies. PRISMA, Preferred Reporting Items for Systematic Reviews and Meta-Analyses

All articles evaluating endoscopic surveillance for dysplasia detection were found by searching PubMed, Science Direct, and Google Scholar. RevMan 5.4 was used for statistical analysis. Following further revision, a total of 13 articles were included in this systematic review after final screening. This screening includes the removal of review articles as well as articles that are unrelated to the study (Figure 1).

Data Characteristics

The detailed summary of all the included studies is given in Table 3.

**Table 3:** Summary table of all included studies. [19-31]. AIH, autoimmune hepatitis; OLT, orthotopic liver transplantation; ALF, acute liver failure; UDCA, ursodeoxycholic acid; PBC, primary biliary cholangitis; CASP, Critical Appraisal Skills Program; SD, standard deviation; CI, confidence interval; AST, aspartate aminotransferase; ALT, alanine aminotransferase; ALP, alkaline phosphatase; MMF, mycophenolate mofetil; AIC, autoimmune cholangitis

Sr no.	Study	Country	Study design	Patients	Intervention	Main outcomes
1	Purnak et al. (2017) [19]	Turkey	Retrospective cohort	There were 71 AIH patients.	Azathioprine and prednisolone were administered to all patients.	The combination therapy showed fewer relapsing events.
2	Manns and Strassburg (2011) [20]	Germany	Retrospective observational study	AIH	Prednisolone monotherapy and azathioprine and prednisolone combination therapy	Budesonide should not be used in patients with cirrhosis.
3	Chazouillères et al. (2006) [21]	France	Retrospective cohort	17 patients of overlap syndrome	First-line treatment consisted of UDCA, and it was used in combination with immunosuppressants.	For overlap syndrome, a combination of UDCA and immunosuppressors was very effective.
4	Ozaslan et al. (2010) [22]	Turkey	Retrospective study	AIH-primary biliary cholangitis (PBC) and 10 of those with AIH-AIC	Combination and immunosuppressive therapy	Corticosteroids and UDCA were very effective when used in combination.
5	Chazouillères et al. (1998) [23]	Turkey	Retrospective cohort	130 hepatitis B surface antigen-negative patients with a diagnosis of PBC	Corticosteroids	This retrospective study found that in patients with PBC: (1) overlap syndrome with AIH was not uncommon (9%); (2) AIH flares can occur spontaneously or as a result of medication under UDCA therapy; and (3) most patients required a combination of UDCA and corticosteroids to achieve a complete clinical and biochemical response.
6	Tanaka et al. (2011) [24]	Japan	Retrospective study	33 patients with AIH-PBC	Combination therapy	In PBC-AIH overlap, AIH-like features predominated in liver histology.
7	Moura (2014) [25]	Portugal	Retrospective study	Patients with AIH	Cyclophosphamide, UDCA	Other drugs can be used if corticosteroids produce side effects.
8	Pniewska et al. (2016) [26]	Poland	Retrospective study	15 patients with AIH	Treatment with prednisone and azathioprine	Most patients' prognoses improved significantly as a result of the treatment, allowing for biochemical remission of the disease. However, significant treatment side effects pointed to the need for further research into effective and safe therapy, particularly in the pediatric population.
9	Zachou et al. (2011) [27]	Greece	Cohort study	Fifty-nine patients with AIH	Prednisolone and mycophenolate	Mycophenolate appeared to be safe and effective as first-line therapy.
10	Park et al. (2010) [28]	Korea	Retrospective study	158 patients with AIH	UDCA and steroid combination therapy	Patients with AIH-PBC overlap syndrome responded less well to UDCA and steroid combination therapy and progressed more quickly to cirrhosis than patients with AIH.
11	Reichenberger et al. (2006) [29]	Germany	Retrospective cohort	Chronic viral hepatitis, alcoholic liver disease, and AIH	Sildenafil	Finally, sildenafil may be effective in portopulmonary hypertension alone or in combination with inhaled prostanoids.
12	Malaguarnera et al. (2004) [30]	Italy	Randomized controlled study	The study included 42 patients with chronic hepatitis C and a history of autoimmune disease.	Intravenous immunoglobulin plus interferon-α	Immunoglobulins helped in the treatment of hepatitis.
13	Friedlander et al. (2019) [31]	Australia	Retrospective cohort	49 patients of AIH	Pamiparib or tislelizumab	Continued the trial and investigated the activity of pamiparib plus tislelizumab in disease-specific cohorts.

CASP Assessment

To evaluate the methodological quality of the studies included in the meta-analysis, a quality assessment table, designated as Table 4, was created using the Critical Appraisal Skills Program (CASP) tool. The evaluation was based on a modified version of the CASP criteria, which were first developed in 1993 by Guyatt et al. [32] (Table 4).

**Table 4:** CASP (modified low appraisal criteria) analysis. Y, Yes; N, No; ?, Can’t tell

Questions	Purnak et al. (2017) [19]	Manns and Strassburg (2011) [20]	Ozaslan et al. (2010) [22]	Chazouillères et al. (1998) [23]	Tanaka et al. (2011) [24]	Moura (2014) [25]	Pniewska et al. (2016) [26]	Zachou et al. (2011) [27]	Park et al. (2010) [28]	Reichenberger et al. (2006) [29]	Malaguarnera et al. (2004) [30]	Friedlander et al. (2019) [31]
Did the study address a clearly focused question?	Y	Y	Y	Y	Y	Y	Y	Y	Y	Y	Y	Y
Did the authors look for the right type of papers?	Y	Y	Y	Y	Y	Y	Y	Y	Y	Y	Y	Y
Do you think all the important, relevant studies were included?	Y	Y	?	Y	Y	Y	?	N	Y	Y	Y	Y
Did the review’s authors do enough to assess the quality of the included studies?	Y	?	N	N	Y	Y	?	N	N	Y	Y	Y
If the results of the review have been combined, was it reasonable to do so?	Y	Y	?	N	N	Y	N	N	Y	N	N	N
Have the authors taken account of the potential confounding factors in the design and/or in their analysis?	?	N	Y	N	N	Y	N	N	N	Y	Y	Y
Are the results clear to the reader?	Y	Y	Y	Y	N	?	Y	Y	Y	Y	Y	Y
Are the results precise?	Y	?	Y	Y	N	N	N	Y	N	Y	Y	Y
Is the model validated?	?	Y	?	N	Y	N	N	Y	N	Y	Y	Y
Is the model applicable to a local population?	N	?	?	N	Y	Y	N	Y	N	Y	Y	N
Do the results fit with other available evidence?	Y	Y	Y	Y	Y	Y	Y	Y	Y	Y	Y	Y
Score out of 11	8	7	6	6	7	8	4	7	6	10	10	9

Meta-Analysis

Meta-analysis was done for the continuous variables using mean, standard deviation (SD), and sample size When these specific data were unavailable, the means and SDs of the intervention and control groups' outcome measurements were used as a substitute. Every study with relevant outcome data was included, and primary analyses were performed for each score. RevMan 5.4 from the Cochrane database was used.

**Figure 2:**
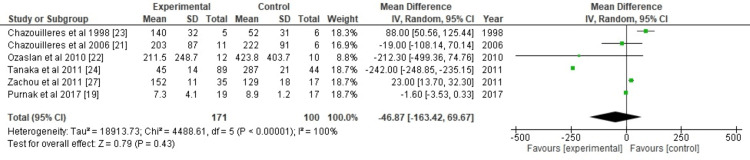
Forest plot of ALT. [19,21,22,23,24,27]. SD, standard deviation; CI, confidence interval; ALT, alanine aminotransferase

ALT: To collect continuous data, a forest plot was created for each of the six different studies. The overall effect size was calculated using Cohen's *d*, with *d *= -46.87, 95% confidence interval (CI) = -163.42 to 69.67. The calculated heterogeneities were Chi^2 ^= 4488.61, degrees of freedom (df) = 5 (*P*-value = 0.00001), and *I*^2 ^= 100%. After analysis, the overall effect was determined to be *Z *= 0.79 (*P *= 0.43). Individual effects favored the experimental group over the control group in each study (Figure 2).

**Figure 3:**
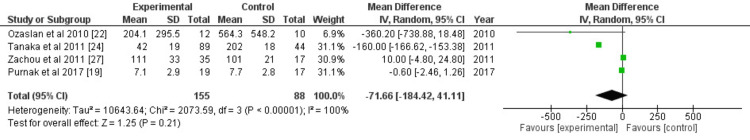
Forest plot of AST. [19,22,24,27]. SD, standard deviation; CI, confidence interval; AST, aspartate aminotransferase

AST: Using continuous data, the forest plot was created for four different studies. The overall effect size was calculated using Cohen's *d*, with *d *= -71.66, and 95% CI = -184.42 to 41.11. The calculated heterogeneity was as follows: *I*^2 ^= 100%, Chi^2 ^= 2073.59, and df = 3. The overall effect analysis, *Z *= 1.25 (*P *= 0.21), was discovered. Individual effects in each study favored the experimental group (Figure 3).

**Figure 4:**
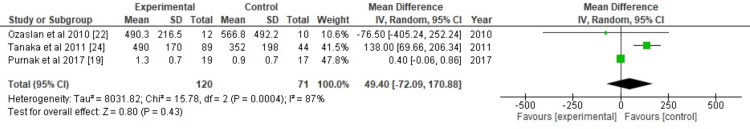
Forest plot of ALP. [19,22,24]. SD, standard deviation; CI, confidence interval; ALP, alkaline phosphatase

ALP: Using continuous data, the forest plot was created for three different studies. The overall effect size was calculated using Cohen's *d*, with *d* = -49.40, and 95% CI -72.09 to 170.88. The calculated heterogeneity was as follows: *I*^2 ^= 87%, Chi^2 ^= 15.78, and df = 2 (*P*-value = 0.0004). The overall effect analysis, *Z *= 0.80 (*P *= 0.43), was discovered. Individual effects in each study favored the experimental group (Figure 4).

Discussion

Alanine aminotransferase (ALT), aspartate aminotransferase (AST), and alkaline phosphatase (ALP) were all evaluated in the meta-analysis. For each variable, forest plots were created separately. In terms of the specified biochemical markers, the results consistently favored the experimental group, indicating that the intervention had a positive impact on ALT, AST, and ALP levels.

The comprehensive analysis of the 13 included studies consistently highlights the strong therapeutic effectiveness of prednisolone and azathioprine combination therapy. This treatment strategy works well not only in the treatment of AIH in isolation but also when AIH coexists with primary biliary cholangitis (PBC). The synthesis of evidence from these studies emphasizes the importance of prednisolone and azathioprine's synergistic action, implying a valuable treatment avenue for clinicians managing patients with AIH, particularly in cases of PBC.

While prednisolone or prednisolone plus azathioprine is effective for 80% of patients, the remaining 20% face challenges such as refractory disease, poor compliance, or drug intolerance, according to a review. According to experts, non-responders and those with intolerance should take high-dose prednisolone with azathioprine. However, new evidence suggests that there may be alternatives, with budesonide promising due to its liver-specific metabolism and lower steroid-related side effects. Mycophenolate mofetil (MMF) is thought to be a promising remission maintenance treatment option, particularly in patients with azathioprine intolerance or refractory AIH [33]. Another review emphasizes the importance of considering alternative strategies, particularly when standard therapy fails to achieve remission or when patients suffer from drug toxicity. These novel approaches are thought to improve treatment outcomes for people with AIH [34].

Another study suggests that patients with pre-symptomatic or symptomatic PBC and AIH should be treated as soon as possible. Ursodeoxycholic acid (UDCA) and corticosteroids are used in the proposed method [35].

While the reviews and studies discussed are useful, there are a few limitations to be aware of. To begin, the studies differ in terms of patient groups, treatments, and study designs. This may introduce bias and make it difficult to generalize the findings. Furthermore, some studies relied on self-reported data, while others examined historical data, which may not be the most reliable method of data collection. The ever-changing landscape of how we treat AIH and PBC may have also gone unnoticed in the studies we looked at. Finally, some studies may not have followed patients for a long enough period of time to determine how well the treatments worked.

## Conclusions

The systematic review and meta-analysis presented a comprehensive evaluation of combination therapies, such as prednisolone and azathioprine, for treating autoimmune hepatitis. The inclusion criteria were meticulous, focusing on studies with specific interventions and measurable outcomes, which were methodically analyzed using RevMan 5.4. Despite the high prevalence and complex clinical profile of AIH, particularly in demographic groups like Alaska Natives, the findings suggest that combination therapies provide significant benefits in managing the disease, reducing recurrence rates, and improving overall patient outcomes. This review underscores the importance of targeted treatment approaches and highlights the need for ongoing research to refine and optimize therapeutic strategies for AIH, addressing both efficacy and safety.
